# An Efficient Acoustic Camera Simulator for Robotic Applications: Design, Implementation, and Validation

**DOI:** 10.3390/s24237835

**Published:** 2024-12-07

**Authors:** Jisung Park

**Affiliations:** Department of Mechanical Convergence Engineering, Gyeongsang National University, Changwon-si 51391, Republic of Korea; jisung@gnu.ac.kr

**Keywords:** forward-looking imaging sonar, acoustic camera, active sonar model, acoustic camera simulator, robotics simulator, underwater robotics applications

## Abstract

As forward-looking imaging sonar, also called an acoustic camera, has emerged as an important sensor for marine robotics applications, its simulators have attracted considerable research attention within the field. This paper presents an acoustic camera simulator that efficiently generates acoustic images using only the depth information of the scene. The simulator approximates the acoustic beam of a real acoustic camera as a set of acoustic rays originating from the center of the acoustic camera. A simplified active sonar model and error models for the acoustic rays are incorporated to account for the environmental factors which can affect the rays. The simulator is implemented in the Gazebo simulator, a robotic simulator, and does not rely on any other components except depth rendering. To evaluate the performance of the developed simulator, qualitative and quantitative comparisons were conducted between simulated images and actual acoustic images obtained in a test tank.

## 1. Introduction

Forward-looking imaging sonar, also called an acoustic camera, is an active sonar system that uses an array of transducers to generate acoustic waves at approximately 1 MHZ. These acoustic waves are focused and radiated within a specific range of altitude and azimuth, creating a two-dimensional image based on the intensity of the received echoes. This sensor is widely used in various underwater applications, as it can produce two-dimensional imaging results for underwater environments similar to those from an optical camera.

Acoustic cameras are one of the few imaging sensors that can be used in underwater environments and have attracted significant research attention in underwater robotics applications. However, underwater robotics research using acoustic cameras has primarily been conducted by a few number of institutions and universities due to the high cost of robotic platforms and acoustic cameras, the difficulty of accessing real sea environments, and the expense of building or using test tanks. For this reason, there has been increasing interest in developing simulators for acoustic cameras.

Simulating the signals of acoustic sensors typically requires modeling both the propagation of acoustic waves and the effects of various environmental factors, making it a complex and computationally intensive process. Fortunately, acoustic cameras have a relatively low signal-to-noise ratio and short detection range compared to other acoustic sensors, allowing certain processes in acoustic image simulation to be simplified. Accordingly, an acoustic camera simulator developed with these considerations in mind would be highly efficient and could be effectively utilized in underwater robotics applications.

With advancements in sonar technology, underwater sonar devices are now used in a variety of marine robotics applications, including target recognition, localization, mapping, and simultaneous localization and mapping (SLAM). Consequently, several sonar simulators have been developed for academic research purposes, and these can be categorized into three main types of simulation techniques for sonar imaging [1].

Frequency domain models use the Fourier transform of transmitted and received sound pulses [2]. Finite difference models solve the acoustic wave equation [3], numerically determining acoustic pressure, although this approach is computationally intensive. Ray tracing is a rendering technique that can produce high-quality images but requires substantial computational resources due to the complexity of tracing light paths.

Some techniques were presented for simulating side-scan sonar images [4,5]. This technique is based on ray tracing, allowing it to generate realistic sonar data, but at a high computational cost. The method accounts for transducer motion and directivity characteristics, the refractive effects of seawater, and scattering from the seabed to produce synthetic side-scan images. Given the computational demands of ray tracing, some efforts have been made to find alternatives. One such alternative, called tube tracing, was proposed by [6]. Tube tracing uses multiple rays to form a footprint on a detected boundary, requiring less computation than ray tracing. The effectiveness of tube tracing for forward-looking sonar simulations was demonstrated in [7], where object irregularities and reverberation effects are also considered.

A forward-looking sonar simulator based on ray tracing was developed [8]. This simulator uses distance information to the point where each ray intersects an object to generate a sonar image, though it does not incorporate an acoustic wave propagation model, resulting in relatively simple sonar images. Another approach to forward-looking imaging sonar simulation involves using incidence angle information at ray-object intersection points [9]. Ray tracing is combined with a frequency domain method [1].

A forward-looking sonar simulator was developed using the Gazebo simulator and the Robot Operating System (ROS) [10,11]. The simulator generates a point cloud through ray tracing in the Gazebo environment, which is then converted into a sonar image. To produce realistic sonar images, this approach considers object reflectivity as signal strength and applies various image processing techniques.

A GPU-based sonar simulation was proposed in [12], capable of producing images for two types of acoustic cameras: forward-looking sonar and mechanically scanned imaging sonar. This simulator uses ray distance and incidence angle information, but does not account for wave propagation and reverberation effects. A sonar simulator that incorporates the physical properties of acoustic waves was proposed in [13], treating acoustic wave propagation as an active sonar model and computing the received echo-to-noise ratio based on transmitted signal level, transmission loss, noise level, sensor directivity, and target strength.

A sonar simulator that combines ray tracing with style transfer based on a Generative Adversarial Network (GAN) was proposed in [14]. This simulator generates the sonar image using ray tracing, and then a GAN-based style transfer produces realistic sonar images from the simulation images. A point-based scattering model was introduced to simulate the interaction of sound waves with targets and their surrounding environment [15]. While simplifying the complexity of target scattering, the model successfully generates coherent image speckle and replicates the point spread function. This approach, implemented within the Gazebo simulator, also demonstrates that GPU processing can significantly improve the image refresh rate.

The simulators introduced above have primarily focused on the generation of realistic acoustic images, which often requires sophisticated acoustic modeling and GPU-based computations. Fortunately, acoustic camera simulators designed for this purpose can be increasingly important tools in simulations for underwater robotics applications. Meanwhile, since the simulation results of acoustic images can be utilized in underwater robotic applications, such as underwater localization and manipulation, the development of an efficient acoustic camera simulator capable of running on the onboard computing devices of mobile robots has also become essential. Therefore, an acoustic camera designed for this purpose should be able to operate even on low-spec computing devices and should avoid using GPU processing that consumes a significant amount of power. In response to this need, this paper proposes a simulator that can efficiently generate acoustic images without GPU-based computation except for scene rendering.

Specifically, the proposed simulator approximates acoustic beam as a set of acoustic rays using ray-casting engine efficiently. Then, an error model for the acoustic rays is employed to generate realistic acoustic images. This paper is organized as follows. Section 2 describes the imaging geometry of acoustic cameras. Section 3 and Section 4 presents the methods for simulating acoustic images. Section 5 and Section 6 discuss the results of extensive experiments conducted to evaluate the effectiveness and performance of the developed simulator, followed by a discussion of potential future directions. Finally, Section 7 presents the conclusions.

## 2. Imaging Geometry of Acoustic Camera

An acoustic camera emits sound waves in a specific beamforming pattern, determined by the wave frequency and an array of transducers. The beamforming directs acoustic waves to concentrate within a specified range of azimuth and elevation angles. Consequently, the space through which the acoustic waves propagate can be approximated as a fan-shaped region with specific azimuth and elevation angles and a maximum detectable range.

The acoustic camera can detect a large area at once using its transducer array; however, it can not determine the height from which the acoustic waves is reflected. The only information available is the range and azimuth of the point where the acoustic waves are reflected.

**Figure 1:**
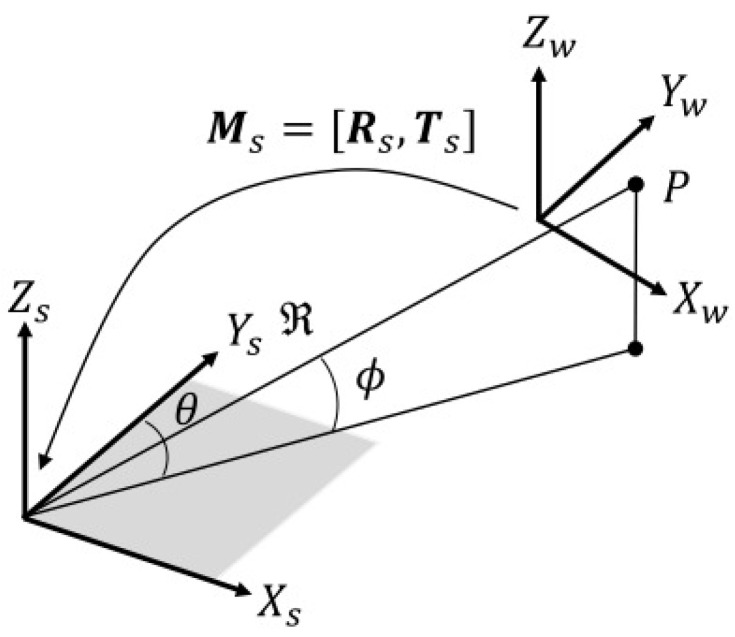
World (or object) coordinate system and acoustic camera coordinate system.

Following the notation in [16], a 3-D point P can be represented in two coordinate systems: rectangular coordinates, ${\left[X,Y,Z\right]}^{T}$, and spherical coordinates, ${\left[R,\phi ,\theta \right]}^{T}$, in the object or world coordinate systems. The point is also considered as a point where an acoustic wave is reflected (see Figure 1).

Let $P_{s}={\left[X_{s},Y_{s},Z_{s}\right]}^{T}$ denote the coordinates of a 3-D point in the acoustic camera coordinate system. The transformation between rectangular and spherical coordinates is as follows:

(1) $$\begin{array}{c} R=\sqrt{X_{s}^{2}+Y_{s}^{2}+Z_{s}^{2}} \end{array}$$

(2) $$\begin{array}{c} \theta =\tan^{-1}\left(\frac{X_{s}}{Y_{s}}\right) \end{array}$$

(3) $$\begin{array}{c} \phi =\tan^{-1}\left(\frac{Z_{s}}{\sqrt{X_{s}+Y_{s}}}\right) \end{array}$$

The transformation matrix $M_{s}$, consisting of the rotation matrix $R_{s}$ and translation vector $T_{s}$, represents the transformation between the world (or object) coordinate system and the acoustic camera coordinate system.

**Figure 2:**
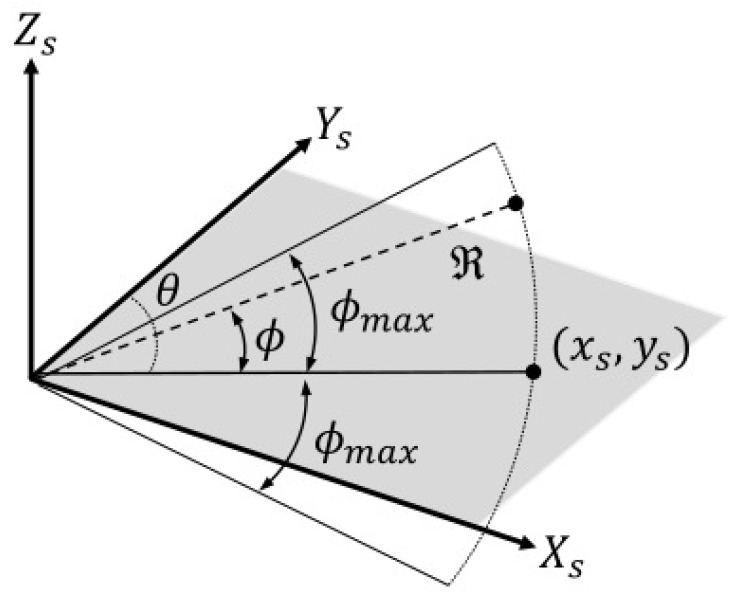
Acoustic camera coordinate system.

In the acoustic camera coordinate system, a point on the image plane that is coplanar with the plane formed by the *x*- and *y*-axes of the acoustic camera coordinate system is determined solely by the range and azimuth angle (see Figure 2), where $\phi_{max}$ is the maximum elevation angle at which acoustic waves can be radiated from the acoustic camera.

The acoustic image is represented in two different coordinate systems: one with symmetric units in meter and another with asymmetric units consisting of meter and azimuth angle. Let $I_{S(x-y)}$ and $I_{S(r-\theta )}$ denote the acoustic image in the symmetric coordinate system and the acoustic image in the asymmetric coordinate system, respectively. Thus, an image point on the acoustic image $p_{S}$ can be expressed as $p_{S}={\left[x_{S},y_{S}\right]}^{T}$ for the acoustic image $I_{S(x-y)}$, or as $p_{S}={\left[R,\theta \right]}^{T}$ for the acoustic image $I_{S(r-\theta )}$. The transformation of two coordinates is given as follows:

(4) $$\begin{array}{c} p_{s}={\left[x_{s},y_{s}\right]}^{T}={\left[R\sin\theta ,R\cos\theta \right]}^{T}, \end{array}$$

where the discrete image coordinates in the two forms of the acoustic image are determined based on the resolution of each image.

## 3. Simulation of Acoustic Camera Image

### 3.1. Acoustic Beam Approximation Using Depth Camera Frustum

**Figure 3:**
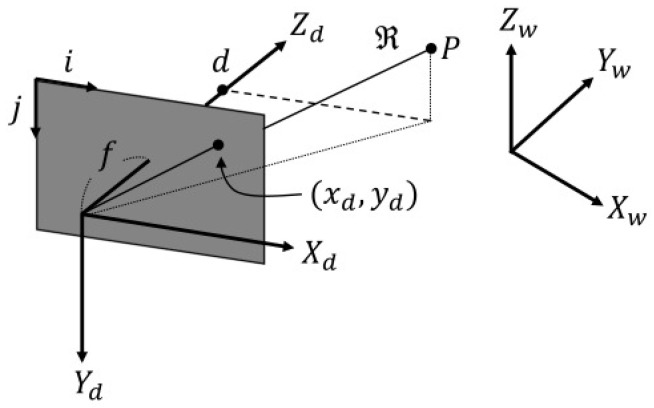
Depth camera coordinate system and its image plane.

The simulation of an acoustic camera image begins by identifying areas in a three-dimensional virtual space where acoustic waves are reflected. A depth camera simulator based on ray-casting is used to obtain a 3-D point cloud that approximates the area where the acoustic waves are incident. Figure 3 shows the coordinate system of a depth camera in the simulator. Here, the axis $Y_{s}$ of the acoustic camera is aligned with the axis $Z_{d}$ of the depth camera, while the axis $X_{s}$ of the acoustic camera is aligned with the axis $X_{d}$ of the depth camera.

Given the depth information from a depth image, it is possible to obtain the range and azimuth information necessary to generate the corresponding acoustic image. Let $I_{d}$ denote a depth image, and let d(i,j) represent the depth at pixel coordinates (i,j) on the image. Let $P_{d}={\left[X_{d},Y_{d},Z_{d}\right]}^{T}$ denote a 3-D point in the depth camera coordinate system. $p_{d(i,j)}={\left[x_{d(i,j)},\;y_{d(i,j)}\right]}^{T}$ denotes a 2-D point corresponding to a pixel coordinates (i,j) on the image, and the 3-D point corresponding to the depth value d(i,j) can be represented as

(5) $$\begin{array}{c} \begin{array}{c} \begin{array}{c} P_{d(i,j)}={\left[\frac{\left(i-c_{x}\right)d\left(i,j\right)}{f_{x}},\frac{\left(j-c_{y}\right)d\left(i,j\right)}{f_{y}},d\left(i,j\right)\right]}^{T}={\left[X_{d(i,j)},Y_{d(i,j)},Z_{d(i,j)}\right]}^{T}, \end{array} \end{array} \end{array}$$

where $c_{x}$ and $c_{y}$ are the coordinates of the principal point of the image plane, and $f_{x}$ and $f_{y}$ are the focal lengths of the depth camera, expressed in pixels.

$P_{d(i,j)}$ is considered as a ray vector originating from the center of the camera and extending to the 3-D point corresponding to the depth value d(i,j). Thus, the range corresponding to the depth d(i,j) can be expressed as

(6) $$\begin{array}{c} \begin{array}{c} R_{i,j}=\sqrt{X_{d(i,j)}^{2}+Y_{d(i,j)}^{2}+Z_{d(i,j)}^{2}} \end{array} \end{array}$$

The azimuth angle can be obtained with the coordinates of the pixel coordinates of the depth image, and it can be represented as

(7) $$\begin{array}{c} \begin{array}{c} \theta_{i,j}=\tan^{-1}\left(\frac{X_{d(i,j)}}{Z_{d(i,j)}}\right)\approx \tan^{-1}\left(\frac{i-c_{x}}{f_{x}}\right), \end{array} \end{array}$$

where $\theta_{i,j}$ denotes the azimuth angle corresponding to the depth d(i,j) and $f_{x}$ is the focal lengths of the depth camera, expressed in pixels, along the *X*-axis of the camera. The surface normal of the 3-D point $P_{d(i,j)}$ can be calculated as

(8) $$\begin{array}{c} \begin{array}{c} n_{i,j}=\left(P_{d(i,j+k)}-P_{d(i,j)}\right)\times \left(P_{d(i+k,j)}-P_{d(i,j)}\right), \end{array} \end{array}$$

where *k* is a parameter, typically set to 2 or 3, which has shown better results in calculating the angle of incidence. With the surface normal, an incident angle corresponding to the depth d(i,j) can be calculated as

(9) $$\begin{array}{c} \begin{array}{c} \delta_{i,j}=\arccos\left(\frac{-P_{d(i,j)}\;\cdot \;n_{d(i,j)}}{|-P_{d(i,j)}\left|\right|n_{d(i,j)}|}\right). \end{array} \end{array}$$

### 3.2. Echo Strength Modeling for a Single Acoustic Ray

The echo strength at each 3-D point determines the pixel intensity in the acoustic image. A real acoustic camera generates acoustic pressure and measures the resulting echo, which is affected by various environmental factors. If the acoustic pressure is accurately modeled, it is possible to create a realistic acoustic image.

The active sonar equation is a widely used model for quantifying acoustic pressure, accounting for various environmental factors that influence the acoustic waves emitted by the acoustic sensors [17]. This equation calculates the received echo-to-noise ratio in decibels. In the equation, the echo-to-noise ratio is determined by combining several environmental parameters, including transmission loss, target strength, noise level, reverberation, detection threshold, array gain, and directivity index (see Equation (10) and Table 1).

(10) SNR=SL−2TL+TS−NL+AG

In the sonar equation, accounting for all environmental factors is challenging and inefficient. However, a sufficiently realistic acoustic image can be achieved by considering only the dominant factors, which offers a more practical and efficient approach. In this analysis, only two dominant factors are considered: transmission loss and target strength. The resulting sonar equation is as follows:

(11) SNR=SL−2TL+TS

Following the notation in [17], the transmission loss is defined as

(12) $$\begin{array}{c} \begin{array}{c} TL=-10\log_{10}\left(\frac{I(R)}{I_{0}}\right)=-20\log_{10}\left(\frac{\left|p(R)\right|}{|p_{0}|}\right), \end{array} \end{array}$$

where I(R) and p(R) denote the acoustic intensity and pressure at a distance *R*, respectively, while $I_{0}$ and $p_{0}$ represent the reference acoustic intensity and pressure. Acoustic waves undergo two types of attenuation as they propagate: spreading loss and absorption in seawater. This relationship can be expressed as

(13) $$\begin{array}{cc} TL & =\mathrm{Spreading}\;\mathrm{Loss}+\mathrm{Absorption}\;\mathrm{in}\;\mathrm{Seawater}. \end{array}$$

The first type of attenuation arises from the geometric spreading of acoustic waves. In a homogeneous medium, acoustic waves propagate spherically outward from the sound source and the intensity at a given point is inversely proportional to the surface area of the expanding sphere. According to the principle of energy conservation, the product of intensity and surface area at any given range must remain constant, as expressed by

(14) $$\begin{array}{c} I\left(R\right)4\pi R^{2}=I_{0}4\pi R_{0}^{2}. \end{array}$$

Consequently, the spreading loss is represented as

(15) $$\begin{array}{c} \begin{array}{cc} TL_{SL}\; & =-10\log_{10}\left(\frac{I(R)}{I_{0}}\right)\\ \; & =-10\log_{10}\left(\frac{4\pi R_{0}^{2}}{4\pi R^{2}}\right)\\ \; & =-20\log_{10}\left(\frac{R_{0}}{R}\right)\\ \; & =20\log_{10}R. \end{array} \end{array}$$

Acoustic waves lose energy through absorption in the medium. In seawater, they interact chemically with boric acid and magnesium sulfate, converting acoustic energy into thermal energy. This form of attenuation depends on the distance the acoustic wave travels, as well as the temperature, pressure, and frequency of the wave. One-way attenuation can be quantified using the spreading distance and the absorptive attenuation coefficient, as follows:

(16) $$\begin{array}{c} TL_{AB}=\alpha R \end{array}$$

where *R* is the distance traveled by the acoustic wave and α is the absorptive attenuation coefficient, given by

(17) $$\begin{array}{c} \alpha =0.106\frac{f_{1}f^{2}}{f_{1}^{2}+f^{2}}e^{\left(\frac{pH-8}{0.56}\right)}+0.52\left(1+\frac{T}{43}\right)\left(\frac{S}{35}\right)\frac{f_{2}f^{2}}{f_{2}^{2}+f^{2}}e^{\left(-\frac{D}{6}\right)}+0.00049f^{2}e^{-\left(\frac{T}{27}+\frac{D}{17}\right)} \end{array}$$

with parameters summarized in Table 2.

The one-way transmission loss is represented by the sum of spreading loss and absorption in seawater, given by

(18) $$\begin{array}{c} TL=TL_{SL}+TL_{AB}. \end{array}$$

Assuming that the reflected acoustic waves spread spherically, the two-way attenuation is represented as 2TL in a homogeneous medium. In the sonar equation, target strength refers to the ability of an object to return an echo. The target strength is defined as the base-10 logarithm of the ratio of the incident acoustic intensity to the reflected acoustic intensity, referenced to a specified distance (typically 1 m) from the object.

The Generic Seafloor Acoustic Backscatter (GSAB) model is employed to represent backscattering strength. To account for the smooth transition between the specular and Lambert modes, a second Gaussian term is included in the model, given by

(19) $$\begin{array}{c} \begin{array}{c} GSAB=Ae^{\left(-\frac{\delta^{2}}{2B^{2}}\right)}+C\cos^{D}\delta +Ee^{\left(-\frac{\delta^{2}}{2F^{2}}\right)}, \end{array} \end{array}$$

where the first two terms represent specular reflection and Lambert’s law for high incidence angles, respectively. The third term describes a transitional level using a Gaussian function. The parameters in the backscattering model are summarized in Table 3.

In Table 3, *A* has a high value for smooth sediment interfaces, *B* represents the half-width of the specular peak, *C* is proportional to frequency and target roughness, and *D* is high for soft and smooth sediment interfaces. For Lambertian scattering, *D* is typically set to 2. *E* denotes the maximum transitional level, and *F* represents its angular half-extent [18]. Consequently, the resulting target strength is given by

(20) $$\begin{array}{c} TS=10\log_{10}\left(GSAB\right). \end{array}$$

### 3.3. Pixel Intensity Determination

Each ray originating from the depth camera determines the imaging region in the acoustic image that corresponds to the area where the ray is incident. By calculating the intensity of each pixel within the region, the acoustic image can be generated. The intensity of each pixel is related to SNR in Equation (11), typically measured in decibels, of the corresponding rays. Basically, acoustic images have a single-channel color space, and the value of each pixel is proportional to the echo strength of acoustic waves returned at the corresponding azimuth angle and range.

If the decibel value of SNR spans a dynamic range (e.g., 0–100 dB), it is possible to scale the decibel value to map the image pixel value by fitting the decibel value to the desired pixel intensity value in a range (e.g., 0–255 for a 8-bit image or 0–4095 for a 12-bit image). For this purpose, the normalized decibel value for a ray, ranging from 0 to 1, is represented as

(21) $$\begin{array}{c} {SNR}^{\prime }=\frac{SNR-dB_{min}}{dB_{max}-dB_{min}} \end{array}$$

where $dB_{min}$ and $dB_{max}$ are a minimum and maximum possible values, respectively, which serve as parameters in the simulator.

In the ray approximation used in this paper, the value of a pixel on an acoustic image is determined by the sum of the SNRs of all rays originating from the same azimuth angle and reflected at the same distance. Let ${SNR}_{R,\theta }^{\prime }$ denote the sum of the normalized SNRs of all rays that share the same azimuth angle and range, which is represented as

(22) $$\begin{array}{c} {SNR}_{R,\theta }^{\prime }=\sum_{k=1}^{N_{R,\theta }}{SNR}_{k,R,\theta }^{\prime }, \end{array}$$

and the corresponding pixel value can be obtained by

(23) $$\begin{array}{c} I_{S}\left(i,j\right)={SNR}_{R,\theta }^{\prime }\;\cdot \;\frac{▵I}{H_{depth}} \end{array}$$

where ▵I is the desired pixel intensity range, $H_{depth}$ is the number of rows of the depth image, which is the same as the number of rays originating from each azimuth angle of the depth camera.

As the pixel values determined by Equation (23) often lie outside the valid range, a bounding operation must be applied to ensure that $I_{S}\left(i,j\right)$ falls within the acceptable range. Consequently, the resulting pixel intensity can be determined as follows:

(24) $$\begin{array}{c} I_{S}\left(i,j\right)=\min(\max\left(I_{S}\left(i,j\right),0\right),▵I) \end{array}$$

### 3.4. Range and Azimuth Angle Uncertainty

For a realistic simulation of acoustic imaging, it is essential to model the errors in the range and azimuth angle measurements of the acoustic camera. While these uncertainties can arise from various factors, it is assumed that they are primarily influenced by frequency and range. Consequently, the errors in both measurements are represented as additive noise modeled by zero-mean Gaussian distributions, expressed as follows:

(25) $$\begin{array}{c} \Delta R∼N(0,\sigma_{R}^{2}\left(R_{i,j},f\right)), \end{array}$$

and

(26) $$\begin{array}{c} \Delta \theta ∼N(0,\sigma_{\theta }^{2}\left(R_{i,j},f\right)), \end{array}$$

where $R_{i,j}$ is a range corresponding to the depth d(i,j) as expressed in Equation (6), *f* is the operating frequency of the acoustic camera, and $\sigma_{R}\left(R_{i,j},f\right)$ and $\sigma_{\theta }\left(R_{i,j},f\right)$ denote the standard deviations of the distribution, quantifying the spread of the noise.

As the distance between the acoustic camera and the object increases, the signal attenuates, reducing the signal-to-noise ratio (SNR) and increasing the uncertainty in both range and azimuth angle measurements. A higher operating frequency of the acoustic camera reduces uncertainty by improving the resolution of the time-of-flight (ToF) measurement, which directly enhances the accuracy of both range and azimuth angle measurements.

Combining the effects of distance and frequency, the standard deviation of the range and azimuth angle uncertainty can be expressed as:

(27) $$\begin{array}{c} \sigma_{R}\left(R_{i,j},f\right)=\sigma_{R,0}\;\cdot \;\left(1+\frac{R_{i,j}^{2}}{R_{0}^{2}}\right)\;\cdot \;\frac{1}{\sqrt{f/f_{0}}}, \end{array}$$

and

(28) $$\begin{array}{c} \sigma_{\theta }\left(R_{i,j},f\right)=\sigma_{\theta ,0}\;\cdot \;\left(1+\frac{R_{i,j}^{2}}{R_{0}^{2}}\right)\;\cdot \;\frac{1}{\sqrt{f/f_{0}}}, \end{array}$$

where $\sigma_{R,0}$ and $\sigma_{\theta ,0}$ represent the baseline uncertainties at the reference range and frequency, $R_{0}$ denotes the reference range at which signal attenuation becomes significant, and $f_{0}$ is the reference frequency. Consequently, the resulting range and azimuth angle can be expressed as:

(29) $$\begin{array}{c} R_{\mathrm{noisy},i,j}=R_{i,j}+\Delta R, \end{array}$$

and

(30) $$\begin{array}{c} \theta_{\mathrm{noisy},i,j}=\theta_{i,j}+\Delta \theta , \end{array}$$

where $R_{\mathrm{noisy},i,j}$ and $\theta_{\mathrm{noisy},i,j}$ are the noisy versions of Equations (6) and (7), respectively.

## 4. Software Implementation

The simulation of acoustic images requires a rendering engine for ray-casting. For this purpose, we use the Gazebo simulator, which is widely utilized in robotics research and supports both scene rendering and a physics engine. Additionally, the simulator is integrated with the Robot Operating System (ROS), enabling easy deployment in robotic applications.

The acoustic image simulation involves the following steps: constructing a virtual environment with a 3-D model, generating a 3-D point cloud through ray-casting, calculating the echo intensity for each ray connecting the virtual camera position to individual points based on the sonar equation, and rendering a acoustic image using the imaging model.

**Figure 4:**
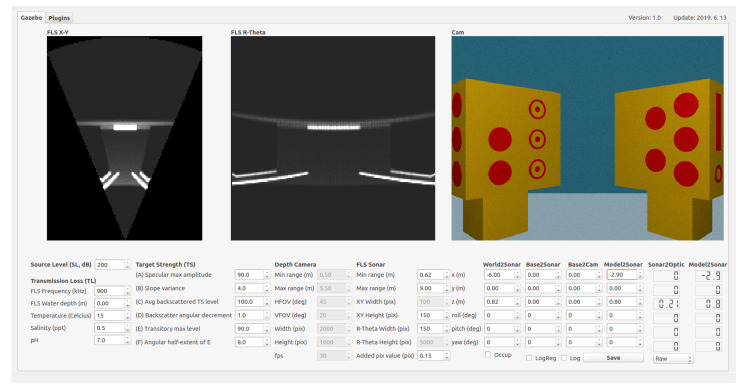
Graphic user interface for acoustic camera simulator.

The 3-D point clouds are generated using a depth camera plugin that leverages the ray-casting engine of the Gazebo simulator. A graphical user interface (GUI) is integrated with the simulator, allowing real-time monitoring of acoustic images and real-time control of all parameters related to the depth camera and sonar equations (see Figure 4). Additionally, various types of noise can be applied to the acoustic images. The overall procedure is summarized in Algorithm 1.   
**Algorithm 1** Acoustic image simulation1 **Input**: $M_{s}=\left(R_{s},T_{s}\right)$▹Figure 1 **Result**: $I_{s(x-y)},\;I_{s(r-\theta )}$ **Parameter:** $k,K_{depth},K_{sonar},K_{attenuation},K_{GSAB}$▹Table 2, Table 3, Table 4
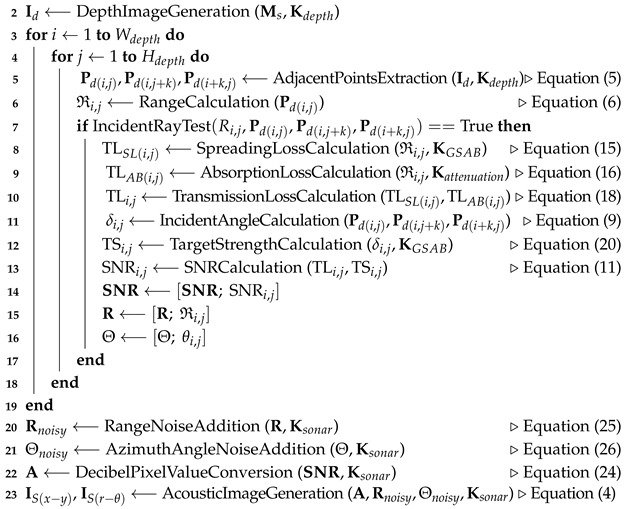


## 5. Simulation Results

**Figure 5:**
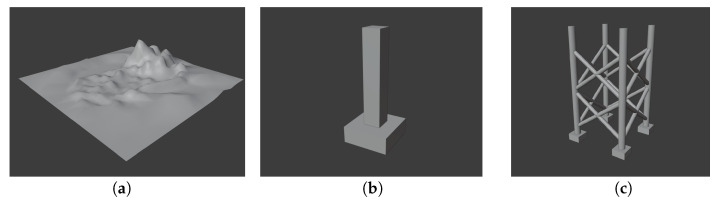
Simulation models: (**a**) Seabed; (**b**) bridge pier; (**c**) jacket.

The performance of the developed simulator was evaluated using three types of models (see Figure 5): a seabed represented as a smooth surface, a bridge pier modeled as a simple polyhedron, and an offshore plant jacket composed primarily of pipe structures.

The dimensions of the three models are as follows: the seabed model has 10 m in length, 10 m in width, and 2 m in height; the bridge pier model has 4 m in length, 4 m in width, and 9.5 m in height; and the jacket model has 5 m in length, 5 m in width, and 8.5 m in height. While the sizes and shapes of these models may differ from their real-world counterparts, this discrepancy does not affect the trends in simulation performance.

**Figure 6:**
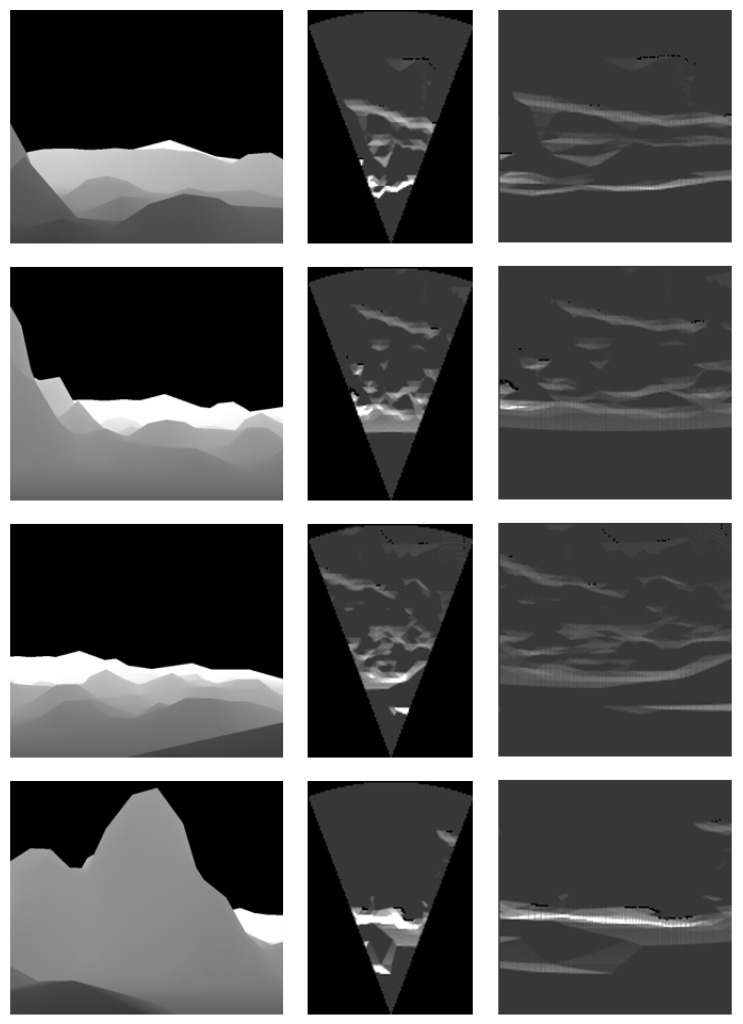
Simulation results for the seabed model.

**Figure 7:**
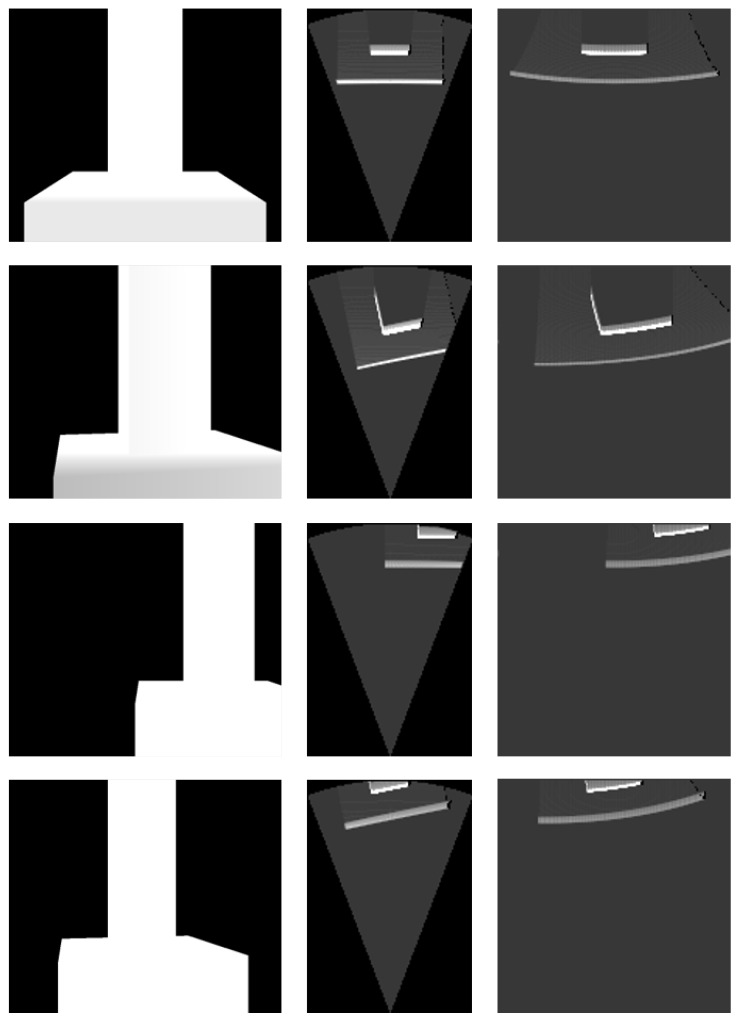
Simulation results for the bridge pier model.

**Figure 8:**
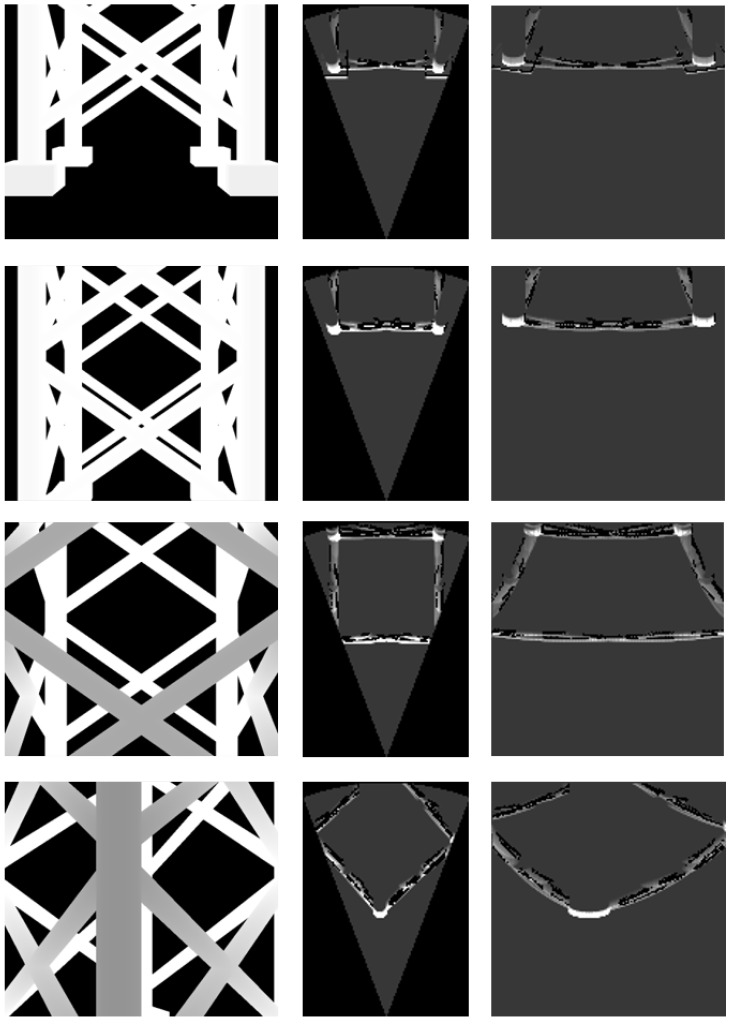
Simulation results for the jacket model.

For each environmental model, acoustic images were generated from several specific locations (see Figure 6, Figure 7 and Figure 8). The simulation parameters were based on the specifications of the Teledyne BlueView P900-45 acoustic camera (Teledyne Technologies, Thousand Oaks, CA, USA), as summarized in Table 4. Since this acoustic camera operates at a relatively low frequency, the images produced by this sensor tend to have low resolution.

Each column in Figure 6 shows a depth image and two types of acoustic images generated by the simulator. The images in each row were obtained from the same camera position in the simulator. In the third column, the images use the horizontal axis to represent azimuth angle and the vertical axis to represent range. The images in the second column align the vertical axis with the Y-axis and the horizontal axis with the X-axis in Figure 1, with both axes measured in meters.

In the acoustic images, a higher pixel value indicates greater echo intensity returned from the corresponding azimuth and range. The simulation results for the seabed model in Figure 6 show the contribution of the sonar equation. Consequently, the simulated images were able to represent echo intensity for terrain with significant elevation changes.

The simulation results for the bridge pier model in Figure 7 demonstrate that the imaging model of the acoustic camera is accurately implemented. The simulated images represent the rectangular shape of the pier without distortion. Likewise, the simulation results for the jacket platform model in Figure 8 show that the the simulator is capable of producing precise images even for complex structure like the jacket platform.

**Table 4:** Parameters for simulating acoustic image of the Teledyne BlueView P900-45.

Parameter	Descriptions
Operating frequency	900 kHz
Operating range	0.62 to 9 m
Source level	200 dB
Water depth	1 to 10 m
Water temperature, salinity, pH	15 °C, 0.5, 7
Target strength (TS) parameters	90 (A), 4 (B), 100 (C), 1 (D), 90 (E), 8 (F)
Depth image resolution	1500 (W) × 1200 (H)
Simulation image resolution	106 (W) × 150 (H)

## 6. Experimental Results

This experimental validation compared acoustic images obtained using the Teledyne BlueView P900-45 acoustic camera with simulated images generated by the acoustic camera simulator. The images were acquired from a model of a well platform, which represents a platform for subsea resource production where underwater robotic applications using acoustic cameras can be applied.

**Figure 9:**
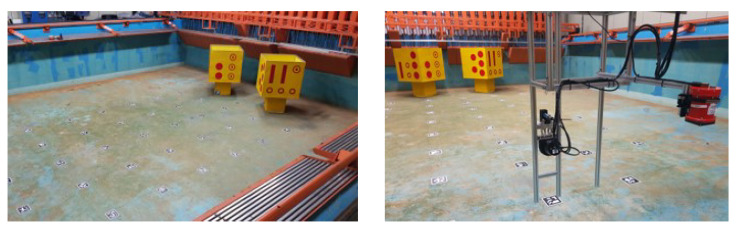
Experimental setup for data acquisition: two mock-up models of a subsea well was placed on a test tank (**left**), and acoustic camera and optical camera were mounted on a frame (**right**).

Acoustic images were captured in a test tank filled with fresh water, allowing for control of experimental conditions (see Figure 9). The tank is rectangular in shape, with dimensions of 15 m in length, 10 m in width, and 2 m in depth. A scaled model of a subsea well was created and placed on the bottom of the test tank. The model is 1 m cube in shape, with a unique pattern on each side to mimic the characteristics of a subsea well.

One acoustic camera and two optical cameras were mounted together on a frame that can move forward, backward, left, and right at a fixed height. The viewing axes of the acoustic camera and one of the optical cameras are parallel to the bottom surface of the test tank. The remaining optical camera was positioned with its viewing axis perpendicular to the bottom of the tank. To determine the positions of the cameras within the tank, artificial visual markers were placed on the bottom and photographed.

**Figure 10:**
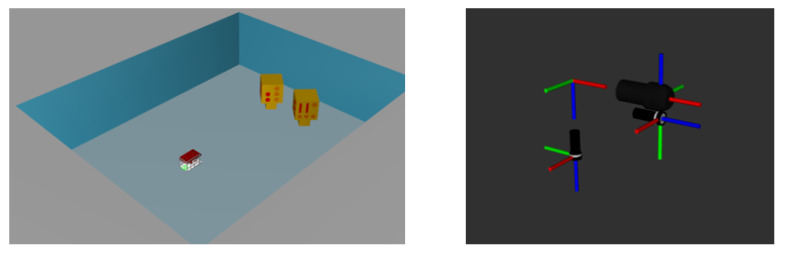
Simulation setup for data acquisition: a CAD model representing the actual mock-up model and test tank was imported (**left**), and arrangement of acoustic camera and optical camera is same as in experimental setup (**right**).

The Gazebo simulator simulated the components and settings of the experimental environment where the actual images were acquired (see Figure 10). The parameters for simulating the acoustic images were set based on the specifications of the actual acoustic camera, the Teledyne BlueView P900-45, summarized in Table 5. The simulated images were acquired at the same location as the actual images, and no acoustic noise was applied.

**Figure 11:**
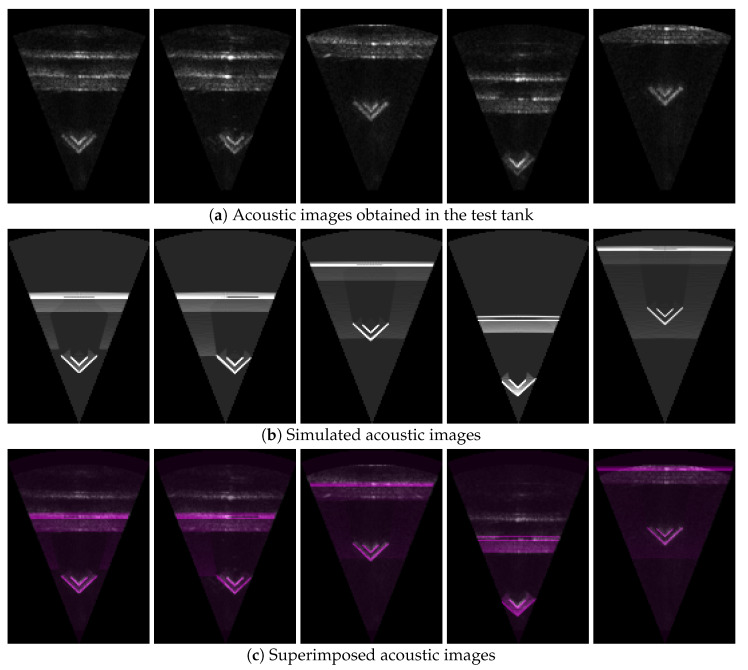
Comparison of acoustic images in the coordinate system shown in Figure 2: (**a**) Acoustic images obtained in the test tank; (**b**) simulated acoustic images; (**c**) superimposed acoustic images.

**Figure 12:**
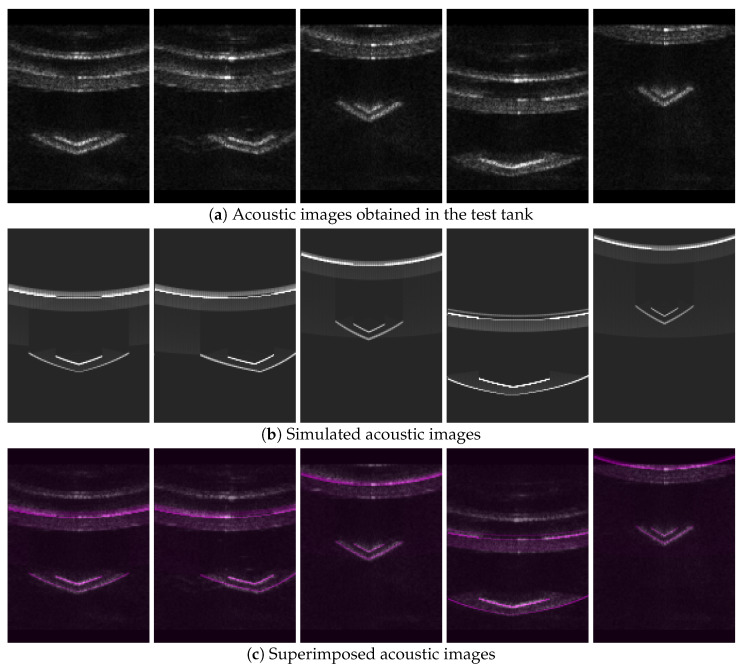
Comparison of acoustic images that use the horizontal axis for azimuth angle and the vertical axis for range: (**a**) Acoustic images obtained in the test tank; (**b**) simulated acoustic images; (**c**) superimposed acoustic images.

Three sets of acoustic images were acquired from the test tank. The real acoustic images suffered from significant noise due to the enclosed environment of the test tank (see Figure 11a and Figure 12a). Consequently, multiple reflections of the acoustic waves were observed in the images, and shadow areas that are typically visible in high-performance acoustic cameras were partially identifiable. The simulated images clearly displayed the shadow areas and were generated at a rate of approximately 15 Hz, which is similar to the actual acoustic camera (see Figure 11b and Figure 12b).

The comparison between real and simulated acoustic images is explained by the overlapping images, target occupancy differences, and pixel intensity differences of the two images. Here, the target occupancy difference refers to the ratio of the region occupied by the object in the simulated image to that in the real acoustic image. To calculate the occupancy difference, the images to be compared are binarized into two parts: the object-occupied region and the background region. Typically, the object regions in the two images differ, primarily due to inaccuracies in image binarization and the CAD model.

The simulated images were overlaid with the real acoustic images for both representations of the acoustic images (see Figure 11c and Figure 12c). The region corresponding to the well platform showed a high level of image alignment. Significant alignment was also observed in the shadow areas created by the well platform. Although multiple reflections from the walls of the test tank contributed considerably to image misalignment, this effect can be disregarded as it is rare in open underwater environments.

**Figure 13:**
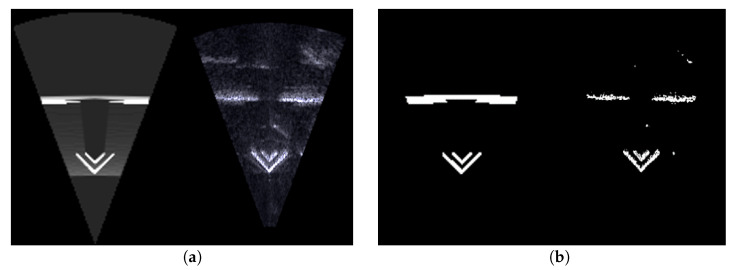
An example of object region comparison. (**a**) Simulated and real acoustic image; (**b**) approximated object regions.

The occupancy differences is evaluated using three metrics (see Table 6): $N_{s}/N_{r}$, $N_{a}/N_{s}$, and $N_{a}/N_{r}$, where $N_{s}$ and $N_{r}$ represent the number of pixels occupied by the object region in the simulated image and in the real acoustic image, respectively (see Figure 13). $N_{a}$ is the number of pixels in the overlap region obtained by the bitwise AND operation of $N_{s}$ and $N_{r}$. These three metrics approach a value of 1 as the two images become more similar.

For all data sets, the real and simulated images showed an occupancy error of approximately 10 to 25 percent. This difference can vary depending on the binarization parameters, so it should be interpreted as an indicator of general trends rather than an absolute measure of difference.

The intensity differences refers to the difference in the pixel value distribution between the two images. This metric indicates how similar the simulated image is to the pixel distribution of the real acoustic image, with a value closer to 0 representing a higher degree of similarity between the images. The two images showed a significant intensity error, primarily due to differences in the imaging modalities (see Table 7). For example, the simulator did not sufficiently account for background noise in the acoustic image, and there was a discrepancy in the parameters related to the material properties of the object.

## 7. Conclusions

Acoustic cameras have a relatively short detection range and focus acoustic waves within a specific range of azimuth and elevation angles to generate acoustic images. These sensor characteristics enable the realistic simulation of acoustic images through ray casting with using an appropriate number of rays and a simplified active sonar model that considers key parameters. The angle of incidence of the acoustic wave on the object is essential information for determining echo intensity in the active sonar model. In this study, a method was employed to calculate the angle of incidence for all rays from the point cloud, enabling the generation of acoustic images with low computing resources and without the need for expensive devices such as GPUs. Consequently, this method can be implemented in any simulator that supports ray casting.

This acoustic camera simulator enables research with acoustic cameras at a low cost. As acoustic cameras are widely used in underwater robotics applications, this simulator is expected to contribute significantly to the advancement of underwater robotics research. One limitation of this study is the absence of direct qualitative and quantitative comparison experiments with other acoustic camera simulators. This is partly due to the limited availability of comparable research conducting such experiments. Future work will address this limitation by performing comprehensive comparisons to further validate the proposed simulator.

## Figures and Tables

**Table 1:** Sonar variables. (Reference intensity is that of a plane wave with the rms pressure level of the reference pressure, usually 1 *μ*Pa).

Variable	Definition
Source level (SL)	10log(intensity at source/at reference)
Transmission loss (TL)	10log(intensity at reference distance/at receiver or target)
Noise level (NL)	10log(intensity of noise/at reference)
Target strength (TS)	10log(intensity at reference distance from target/incident intensity)
Reverberation (RL)	10log(intensity of reverberation at receiver/at reference)
Detection threshold (DT)	10log(signal-to-noise power at output of array)
Array gain (AG)	10log(gain in signal to noise relative to a single omni-hydrophone)
Directivity index (DI)	10log(same as AG but noise is assumed to be uniform in all directions)

**Table 2:** Simulation parameters for the absorptive attenuation coefficient.

Symbol	Parameter	Unit
α	Absorptive attenuation coefficient	dB/m
*f*	Frequency of sonar	kHz
*D*	Water depth	km
*T*	Temperature	°C
$f_{1}$	Relaxation frequency for boric acid	kHz
$f_{2}$	Relaxation frequency for magnesium sulfate	kHz
*R*	Range	km
pH	Hydrogen ion concentration exponent	pH
*S*	Salinity	ppt

**Table 3:** Simulation parameters for the Generic Seafloor Acoustic Backscatter (GSAB) model.

Symbol	Parameter	Unit
δ	Incident angle	degree
*A*	Specular maximum amplitude	-
*B*	Facet slope variance	degree
*C*	Average backscatter level at oblique incidence	-
*D*	Backscatter angular decrement (1 for Lommel–Seeliger, 2 for Lambert)	-
*E*	Transitory maximum amplitude	-
*F*	Angular half-extent of *E*	degree

**Table 5:** Specifications of the acoustic camera, and dimensions of the test tank and mock-up model.

Object	Parameter	Description
**Teledyne BlueView P900-45**	Operating frequency	900 kHz
	Update rate	Up to 15 Hz
	Field of view	45
	Max range	100 m
	Beam width	1–20
	Number of beams	256
	Beam spacing	0.18
	Range resolution	2.54 cm
**Well platform**	Dimensions	1 m × 1 m × 1.5 m (L × W × H)
**Test tank**	Dimensions	12 m × 10 m × 1.5 m (L × W × H)

**Table 6:** Average of occupancy errors.

Data	$N_{s}/N_{r}$	$N_{a}/N_{s}$	$N_{a}/N_{r}$
Dataset 1	1.21	0.75	0.82
Dataset 2	1.10	0.84	0.88
Dataset 3	1.25	0.73	0.80

**Table 7:** Average of intensity errors and simulation time.

Data	X-Y Image	R-θ Image	Simulation Time
Dataset 1	0.28	0.33	0.14
Dataset 2	0.23	0.31	0.18
Dataset 3	0.24	0.30	0.15

## Data Availability

Data is contained within the article.
